# Supporting stroke survivors living in care homes: challenges and opportunities for practice development and research

**DOI:** 10.1093/ageing/afab180

**Published:** 2021-08-28

**Authors:** Jennifer Kirsty Burton, Marion F Walker

**Affiliations:** Institute of Cardiovascular and Medical Sciences, University of Glasgow, Glasgow, Scotland; School of Medicine, University of Nottingham, Nottingham, England

**Keywords:** care home, stroke, data linkage, outcomes, older people

## Key Points

- Stroke survivors moving into care homes have specific needs for their care and support.
- We are still uncertain how best to maintain function or improve the quality of life of care home residents after stroke.
- Data-informed approaches have potential to improve understanding, but must measure outcomes which matter to stroke survivors.
- We require innovative methods and systems, sensitive to the care home context, to enable delivery of the most effective care.

Stroke is recognised to be a life-changing event, which can result in an individual requiring a move to long-term care [1], often termed ‘a care home’ in the United Kingdom (a 24-h care setting with or without on-site registered nursing staff). Those requiring care home admission are typically older, with more severe stroke disease and greater disability [2, 3]. Unfortunately there is limited knowledge about the characteristics, needs and outcomes of this population or how best to deliver targeted services to support them. We therefore welcome the work by Clery *et al.* that uses the South London Stroke Register cohort data to explore survival, health status and care among stroke survivors living in care homes [4]. The methods are novel, using their local stroke register, linking data to electronic primary care records and providing dedicated follow-up using individual assessment or information from family members and care home staff. Such efforts are important as there is a recognised lack of inclusion among those living in care homes in existing UK cohort studies [5].

These data clearly indicate the systematic differences between the populations moving into a care home from their counterparts who can be discharged to their own homes. Striking differences relate to the prevalence of faecal incontinence (56–76% versus 11.5–9.7%), urinary incontinence (70.7–76% versus 11.5–9.7%) and cognitive impairment (69.8–85% versus 36.3–31.4%). The work also quantifies how these have changed over their follow-up period [4], indicating a greater burden of disability among those requiring care home placement over time. Although these data may be unsurprising to stroke clinicians, it is important to explore the factors that differentiate those who are unable to return home after stroke from those who return home successfully.

Residents in care homes have a significantly different life expectancy from those returning home and has been described previously in general population data [6]. However, the improvement in survival after stroke among those returning home not being mirrored among those moving into a care home [4] requires more careful consideration. The clinical data presented show there is increased disability over time [4], concurring with trends recently documented in England and Wales in terms of increased disability and complexity among care home residents [7]. With wider policy directives to enable individuals to remain in their own homes for longer, the outcome is increasingly complex needs on admission to care homes and the consequent impact on the resources and staff required to support such residents.

The challenges of identifying those living in care homes within large clinical and administrative datasets are well-described [8, 9]. but are seen again within this research [4]. There is an urgent need to improve clinical health data systems to accurately record where individuals are admitted from and discharged to, including the ability to identify care home residency on a temporary or permanent basis [10]. This will help us to better understand and evaluate our own clinical services.

Missing data also limit the evaluation of other important outcomes among stroke survivors, including quality of life, anxiety and depression and cognitive performance [4]. The challenges of collecting such data in this care home population highlight the need for more appropriate and feasible measures for those severely impacted by their stroke.

A multicentre cluster trial by Sackley *et al*. [11] evaluated the provision of an occupational therapy intervention targeted at maintaining self-care activities such as washing, dressing, feeding and toileting for residents in care homes. In addition, significant effort focussed on training care home staff to acquire the necessary skills to encourage independence [12]. Despite a neutral outcome, this large study highlighted many key issues such as the lack of autonomy in such a disabled population, the high turnover and resultant lack of continuity of staff who work in this setting, alongside the minefield of who funds equipment to aid independence; the resident, the care home or social care?

It is also reasonable to hypothesise that by improving physical activity in this population, residents may be better able to maintain self-care independence, however a meta-analysis by Crocker *et al*. [13] found the potential for only a very small effect size.

What then are the implications for practice development and research to support stroke survivors living in care homes?

It is apparent from the literature to date that we are still uncertain how best to maintain function or improve the quality of life of care home residents. It is however clear we cannot achieve this by implementing traditional rehabilitation methods. Is it then time we proposed, and evaluated, alternative ways of improving the lives of care home residents? Sensitive and individual environmental design and adaptation may be one way of starting to address the comfort, safety and prevention of complications in this vulnerable group.

Data-informed approaches have the potential to help given the inclusivity and minimal burden these can pose [10]. However, it is necessary to ensure these are capturing measures that are relevant to those living in care homes in terms of their daily lives and measuring outcomes, which are important to them. This may include conditions, such as incontinence or frailty, complications after stroke including those affecting mood and cognition and quality of life measures, which are not collected routinely in health datasets but are highly valued by stroke survivors [14]. These may be richly captured within care home records and care plans, however their use and accessibility outside of the home is limited. There is a desire to develop improved integrated social care and health records, however, we must acknowledge the comparative maturity of routine electronic health data systems in comparison to the secondary use of social care data in the UK context. Data dictionaries, common standards and meanings are a critical underpinning to effective reuse and care must be taken to develop understanding of the origins and purpose behind the original use [15]. If this investment of time and digital infrastructure is not made, there is a risk that social care services, like care homes, are evaluated against health focused metrics (e.g. emergency admission and length of stay) as these are more readily measurable but unlikely to capture the essence of the care being delivered to care home residents.

Stroke survivors moving into care homes have specific needs for their care and support, increasingly more complex than those who can return home. We now urgently require new and innovative methods and systems, sensitive to the care home context, to enable those individuals to receive the most effective care.
